# Acupuncture Attenuates Neuroinflammation Following Acute Cerebral Infarction by Downregulating SPP1 to Ameliorate Immunological Niches

**DOI:** 10.1002/brb3.71699

**Published:** 2026-09-15

**Authors:** Jiang‐Peng Cao, Xiao‐Xi Liu, Ling‐Fei Wang, Ying Zhou, Lin Zhang, Xu‐Juan Chen, Yue‐Xin Lin, Lin‐Ling Chen, Yuan‐Hao Du

**Affiliations:** ^1^ Department of Acupuncture and Moxibustion First Teaching Hospital of Tianjin University of Traditional Chinese Medicine Tianjin China; ^2^ National Clinical Research Center for Chinese Medicine Tianjin China; ^3^ Huzhou Central Hospital Huzhou China

**Keywords:** acupuncture, acute cerebral infarction, immunological niches, neuroinflammation, SPP1

## Abstract

**Background:**

Neuroinflammation following acute cerebral infarction (ACI) critically contributes to disease pathophysiology, with microglia serving as key initial responders. While acupuncture has demonstrated anti‐inflammatory and microglia‐modulating potential, its molecular mechanisms in reshaping immunological niches remain unclear.

**Methods:**

Serum from ACI patients and healthy controls was analyzed using four‐dimensional data‐independent acquisition‐based proteomics to identify differentially expressed proteins (DEPs). The candidate DEPs were validated using enzyme‐linked immunosorbent assay (ELISA), and correlation analyses were performed. We further explored the morphological and molecular biological alterations associated with brain injury and microglial polarization via triphenyltetrazolium chloride (TTC), Nissl, immunofluorescence staining, ELISA, western blot assays, and real‐time quantitative PCR in a rat model of ACI subjected to permanent occlusion of the middle cerebral artery (MCAO) with the treatment of acupuncture.

**Results:**

Acupuncture downregulated elevated SPP1 levels in both MCAO rats and ACI patients. Concomitantly, it substantially improved neurological function, reduced infarction volume, and alleviated neuronal damage in MCAO rats. Additionally, acupuncture promotes polarization of microglia from the M1 to the M2 phenotype, reducing pro‐inflammatory cytokines (IL‐6, IL‐1β, and TNF‐α) and increasing anti‐inflammatory cytokine (Arg‐1, IL‐10, and TGF‐β1). Furthermore, acupuncture may modulate the CD200/CD200R pathway by reducing SPP1 expression.

**Conclusions:**

Acupuncture alleviates brain injury by inhibiting neuroinflammation after MCAO, an effect that might be mediated through the downregulation of SPP1 and the subsequent modulation of CD200/CD200R signaling, potentially restoring immune homeostasis in the ischemic brain.

## Graphical abstract

The study workflow and key mechanistic findings are illustrated. In cohort 1, serum samples from acute cerebral infarction (ACI) patients and healthy controls were screened using proteomics to identify differentially expressed proteins. In cohort 2, the therapeutic effects of acupuncture on ACI were further validated. ELISA and correlation analyses highlighted SPP1 as a central candidate. In a rat model of permanent middle cerebral artery occlusion, acupuncture treatment significantly downregulated SPP1, promoted microglial polarization from the pro‐inflammatory M1 phenotype toward the anti‐inflammatory M2 phenotype, reduced infarct volume, and improved neurological function. These effects were associated with modulation of the CD200/CD200R signaling pathway, suggesting that acupuncture restores immune homeostasis within the ischemic brain immunological niche.

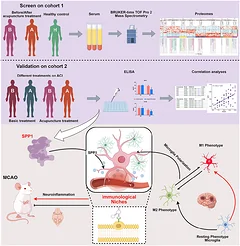

## 1 Introduction

Acute cerebral infarction (ACI) is one of the leading causes of death and disability worldwide (Campbell and Khatri 2020). Cerebral ischemia occurs when the blood flow to brain is disrupted, which causes the brain tissue to become oxygen‐deprived and nutrient‐deprived (Eltzschig and Carmeliet 2011). Current emerging evidences revealed the important role and involvement of inflammation and immune cells invasion within minutes to hours after the initial ischemic lesions (Chamorro et al. 2016; D. Li et al. 2018). As the primary innate immune cells residing in the central nervous system (CNS), microglia respond to cerebral hypoperfusion‐induced brain injury by transitioning along a continuum of functional states. These range from a pro‐inflammatory (classical activation), potentially neurotoxic phenotype, to an anti‐inflammatory (alternative activation), neuroprotective one (Hanisch and Kettenmann 2007). The brain's vascular system is distinctively characterized by the presence of the blood‐brain barrier, which is constituted by the neurovascular unit comprising vascular endothelial cells (VECs), pericytes, astrocytes, microglia, and neurons. Recently, vessel‐associated microglia (VAMs) have been identified in relation to their positioning along blood vessels, although the specific types of blood vessels (capillaries, arterioles, veins, arteries) have not been clearly delineated (Haruwaka et al. 2019). Significantly, the degree of cerebral hypoperfusion in transient ischemia is proportionally reflected in the motility of microglial processes surrounding capillaries, pointing to functions of microglia‐vascular interactions that extend beyond merely reacting to vascular damage. (Masuda et al. 2011). In addition to the extensively studied microglia‐neuron interactions, microglial processes are also recruited to the infarcted vessels, although the precise functions of these interactions remain inadequately defined (Benyó et al. 2016; Zhao et al. 2018). Pathological immunological niches, which are sites of acute inflammation formed by increased oxygen metabolism and the consequent hypoxia following ACI (termed “inflammatory hypoxia”), are closely associated with VECs and may also interact with other cell types, such as microglia (Taylor and Colgan 2017; Aldape et al. 2019). Further research aimed at regulating immunological niches and thereby ameliorating this cerebral ischemia‐induced neuroinflammation is required.

Derived from traditional Chinese medicine, acupuncture therapies such as electroacupuncture and transcutaneous electrical acupoint stimulation are commonly employed as complementary and alternative therapies for various conditions, including ACI (Tsai et al. 2024; Li et al. 2022; L. Chen et al. 2016). Our previous clinical study has revealed that acupuncture intervention is safe and effective in improving the neurological function of patients with ACI (Cao et al. 2025). The results of our preclinical meta‐analysis indicate that acupuncture stimulation has the potential to improve regional cerebral blood flow and alleviate neurological deficits, possibly by regulating vascular smooth muscle cell function (Cao et al. 2024). Furthermore, in our preliminary experiments, we observed that electroacupuncture have a pleiotropic effect and may play different roles in the regulation of vascular endothelial and smooth muscle cells, being pro‐angiogenic in a context‐dependent way in a middle cerebral artery occlusion (MCAO) rat model (Li et al. 2022; J. Li et al. 2014; J. Li et al. 2017; Du et al. 2011). However, the regulatory mechanism of acupuncture underlying the VAMs in the immunological niches are still poorly understood.

Therefore, the aim of this study is to determine whether acupuncture alleviates neuroinflammation after ACI by regulating the interactions of VAMs in the immune niche, and to explore its regulatory mechanism.

## 2 Results

### 2.1 Baseline Characteristics of Participants

A total of 80 participants were enrolled in this study; of these, cohort 1 included 10 healthy control subjects (HC) and 10 patients with ACI participated in this DIA‐based proteomic analysis. The remaining 60 patients with ACI were used in subsequent validation experiments (cohort 2). There were no significant group differences in baseline characteristics of both two cohorts (Tables 1 and 2).

**TABLE 1:** Baseline characteristics of patients with ACI and healthy controls in cohort 1.

Characteristic/groups	ACI patients (*n* = 10)	Healthy controls (*n* = 10)	*p* value^a^
Age, mean (SEM), *y* ^b^	64.10 ± 2.85	62.10 ± 1.75	0.598
Sex, no. (%)^c^			
Female	6 (60.00%)	5 (50.00%)	0.597
Male	4 (40.00%)	5 (50.00%)	
Diagnose	Acute cerebral infarction	NA	NA

Abbreviations: ACI, acute cerebral infarction; NA, not applicable; no. (%), number; SEM, standard error of the mean.

^a^ All tests were two‐sided. Statistical significance was set at *p* < 0.05.

^b^ Analyzed using independent samples *t*‐test.

^c^ Analyzed using chi‐square test.

**TABLE 2:** Baseline characteristics of participants in cohort 2.

Characteristic	Acupuncture group (*n* = 30)	Control group (*n* = 30)
Age, median (IQR), *y*	64.50 [58.00, 68.75]	63.50 [56.00, 70.00]
Female	8 (26.67%)	9 (30.00%)
Male	22 (73.33%)	21 (70.00%)
BMI, median (IQR)^a^	24.34 [22.71, 26.08]	25.51 [23.71, 27.68]
Course of disease, median (IQR), days	2.00 [1.00, 2.00]	1.50 [1.00, 2.00]
Current smoker	21 (70.00%)	13 (43.33%)
Current drinker	13 (43.33%)	9 (30.00%)
Previous stroke	11 (36.67%)	6 (20.00%)
Hypertension	20 (66.67%)	19 (63.33%)
Diabetes	10 (33.33%)	11 (36.67%)
Heart disease^b^	7 (23.33%)	7 (23.33%)
NIHSS score, median (IQR)^c^	11.00 [9.25, 12.00]	11.00 [8.25, 13.00]
FMA score, mean (SD)^d^	48.70 (9.24)	49.40 (6.33)
BI, median (IQR)^e^	50.00 [40.00, 55.00]	45.00 [40.00, 50.00]
mRS, median (IQR)^f^	3.50 [3.00, 4.00]	3.00 [3.00, 4.00]
SPP1, mean (SD)	149.49 (7.95)	151.47 (8.10)
ACAN, median (IQR)	91.70 [74.45, 103.76]	101.70 [70.07, 104.31]

*Note*: Data are presented as number (percentage) of patients unless otherwise.

Abbreviations: BI, Barthel index; BMI, body mass index; FMA, Fugl‐Meyer assessment; mRS, Modified Rankin Scale; NIHSS, National Institute of Health Stroke Scale.

^a^ Body mass index is calculated as weight in kilograms divided by height in meters squared.

^b^ Heart disease was defined as a range of major clinical heart and circulatory disease conditions, including but not limited to rhythm disorders, coronary artery disease, heart failure, and valvular disease.

^c^ Scores range from 0 to 42, with higher scores indicating more serious neurological impairment.

^d^ Scores range from 0 to 100, including upper extremity (FMA‐UE, scores range from 0 to 66) and lower extremity (FMA‐LE, scores range from 0 to 34), with higher scores indicating better motor performance.

^e^ A tool to assess functional independence to perform 10 daily activities, scores range from 0 to 100.

^f^ A global stroke disability scale with scores ranging from 0 (no symptoms or completely recovered) to 6 (death).

### 2.2 Quality Verification of Extracted Protein

Thirty serum samples were collected: 10 from patients prior to acupuncture treatment (BT), 10 from patients after acupuncture treatment (AT), and 10 from healthy controls (HC). Following depletion of high‐abundance proteins, total protein in each serum sample was quantified using the BCA protein assay (Pierce). The samples were then separated by SDS‐PAGE and stained with Coomassie brilliant blue. Quantitative results are presented in Table S1. SDS‐PAGE revealed clear, abundant bands (Figure S1), and high‐abundance protein depletion was confirmed in all samples.

**FIGURE 1:**
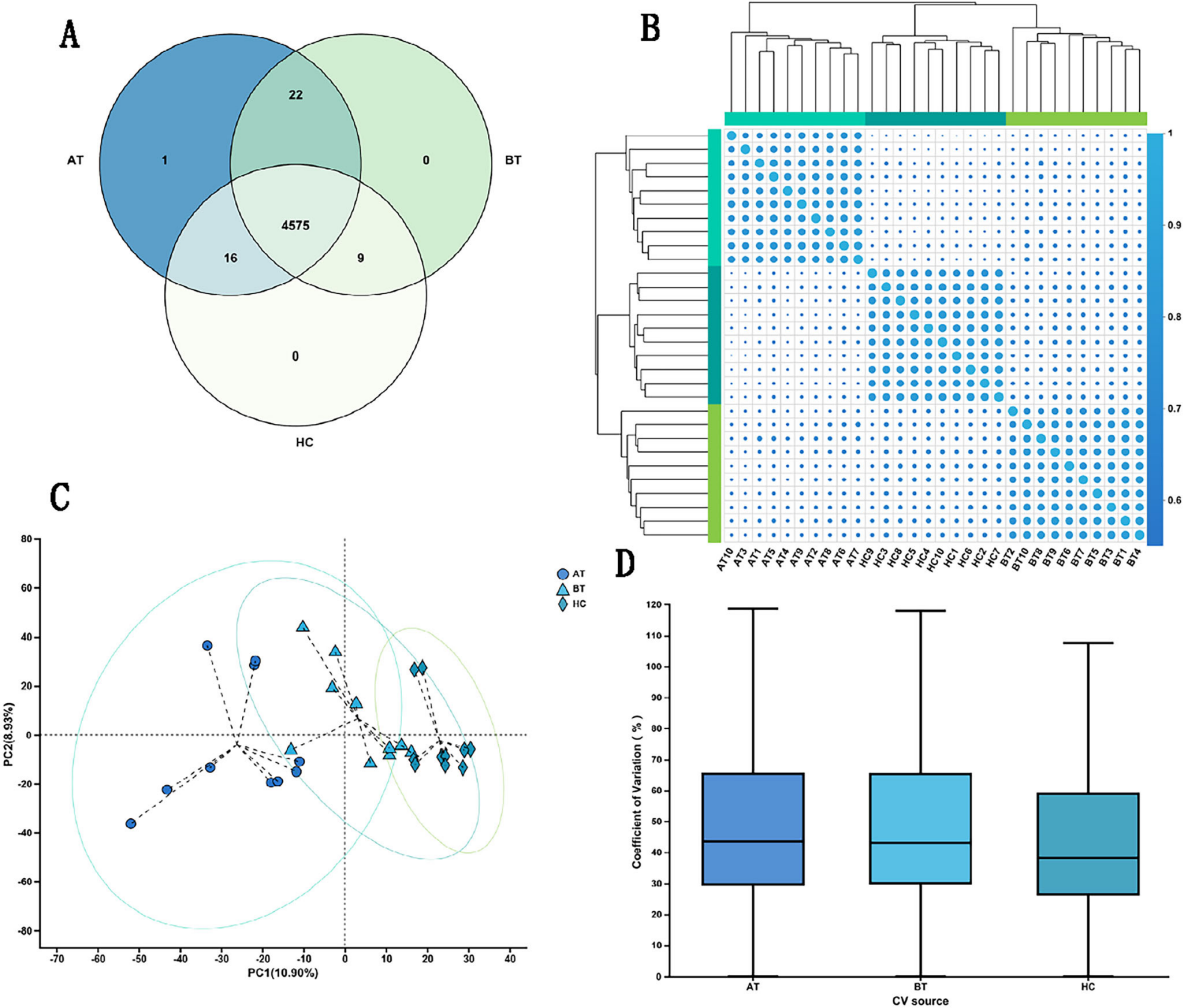
Quality control of proteins in ACI patients and healthy control participants serum. (A) Venn diagram summarized the results of differentially enriched proteins in serum. (B) Heatmap of relative expression of proteins in the serum of three groups. (C) PCA analyses for three groups. (D) Coefficient of variation value distribution for DIA approach.

Data independent acquisition proteomic analysis was used for all depleted serum samples, which were performed using a timsTOF Pro2 high‐resolution mass spectrometer (Bruker, Germany). In the present study, most peptides were between 7 and 27 amino acid residues long (average molecular weight of amino acid residue is 110 Da), indicating that the sample preparation met the technical standard. A total of 5020 proteins were detected, of which Venn diagram showed the identified proteins common among the three groups accounted for 97.65% (Figure 1A). The heatmap of the relationship showed a high correlation of samples with in a group and a low correlation of samples between the groups (Figure 1B). Furthermore, principal component analysis (PCA) was used to analyze the differences among the samples and demonstrated that a distinct homogeneity within a group and a clear difference among groups (Figure 1C,D).

### 2.3 Statistics and Analysis of Differentially Expressed Proteins

**FIGURE 2:**
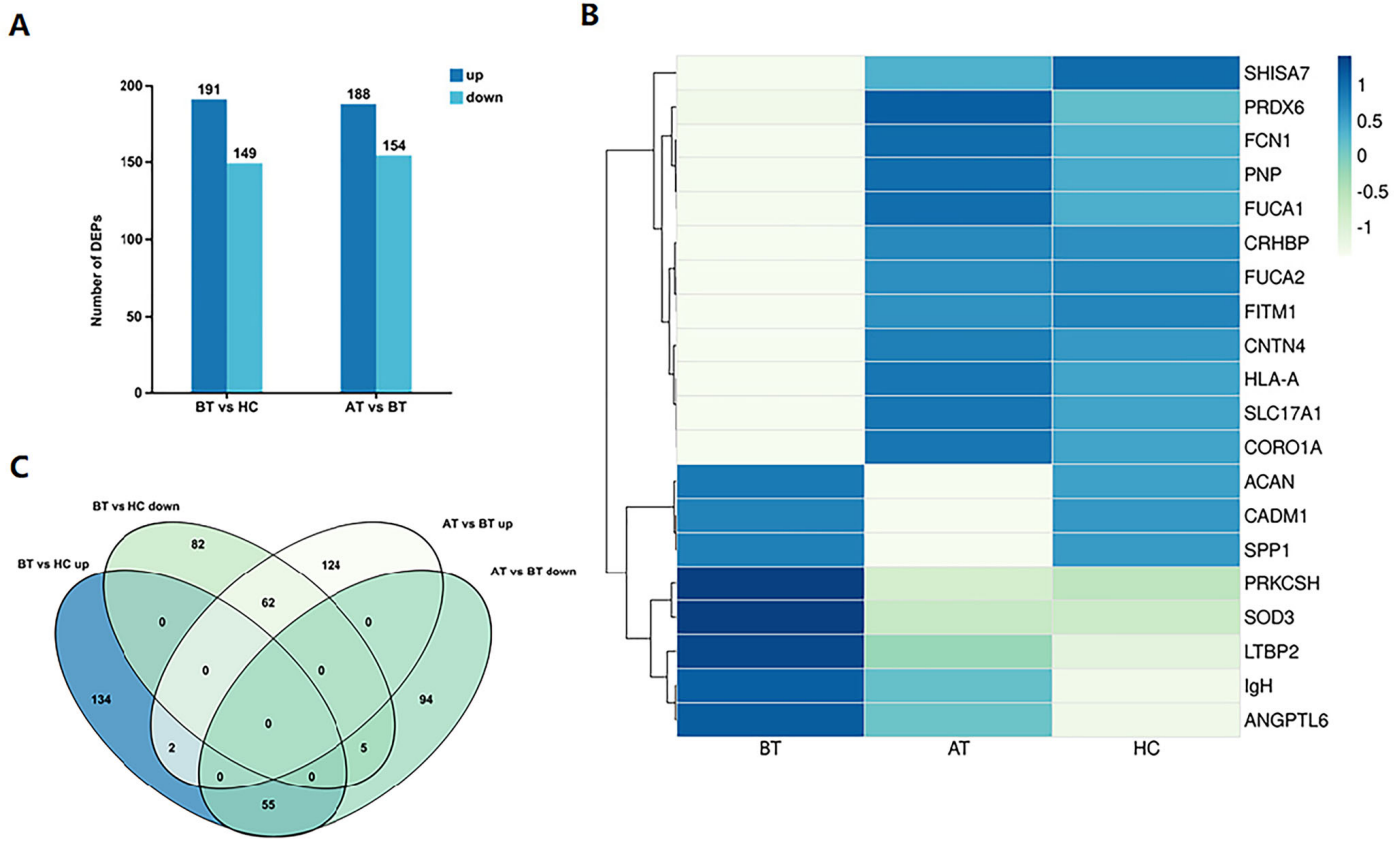
Analysis of differentially expressed proteins in ACI patients and healthy control participants serum. (A) Number of differentially expressed proteins among different groups. (B) Hierarchical clustering heat map showing all detected proteins in the three groups. (C) Venn diagram of intersection analysis.

In this study, differentially expressed proteins (DEPs) between the sets were compared using |log_2_FC|≥ 2 and unadjusted *p* < 0.05. A total of 340 DEPs related to disease were identified by comparing the data of the BT group and the HC group, which included 191 significantly upregulated proteins and 149 significantly downregulated proteins. To investigate the therapeutic mechanism of acupuncture, we compared the DEPs in ACI patients before and after the acupuncture treatment, and there were 342 DEPs, of which, 188 were upregulated proteins and 154 were downregulated proteins (Figure 2A). A hierarchical clustering analysis of DEPs revealed different expression patterns in all three groups (Figure 2B). Further analysis showed that there were 55 common positive proteins when ACI upregulated and acupuncture downregulated, while 62 common positive proteins were found which ACI downregulated and acupuncture upregulated (Figure 2C).

### 2.4 GO and KEGG Analysis of Disease‐Associated DEPs

**FIGURE 3:**
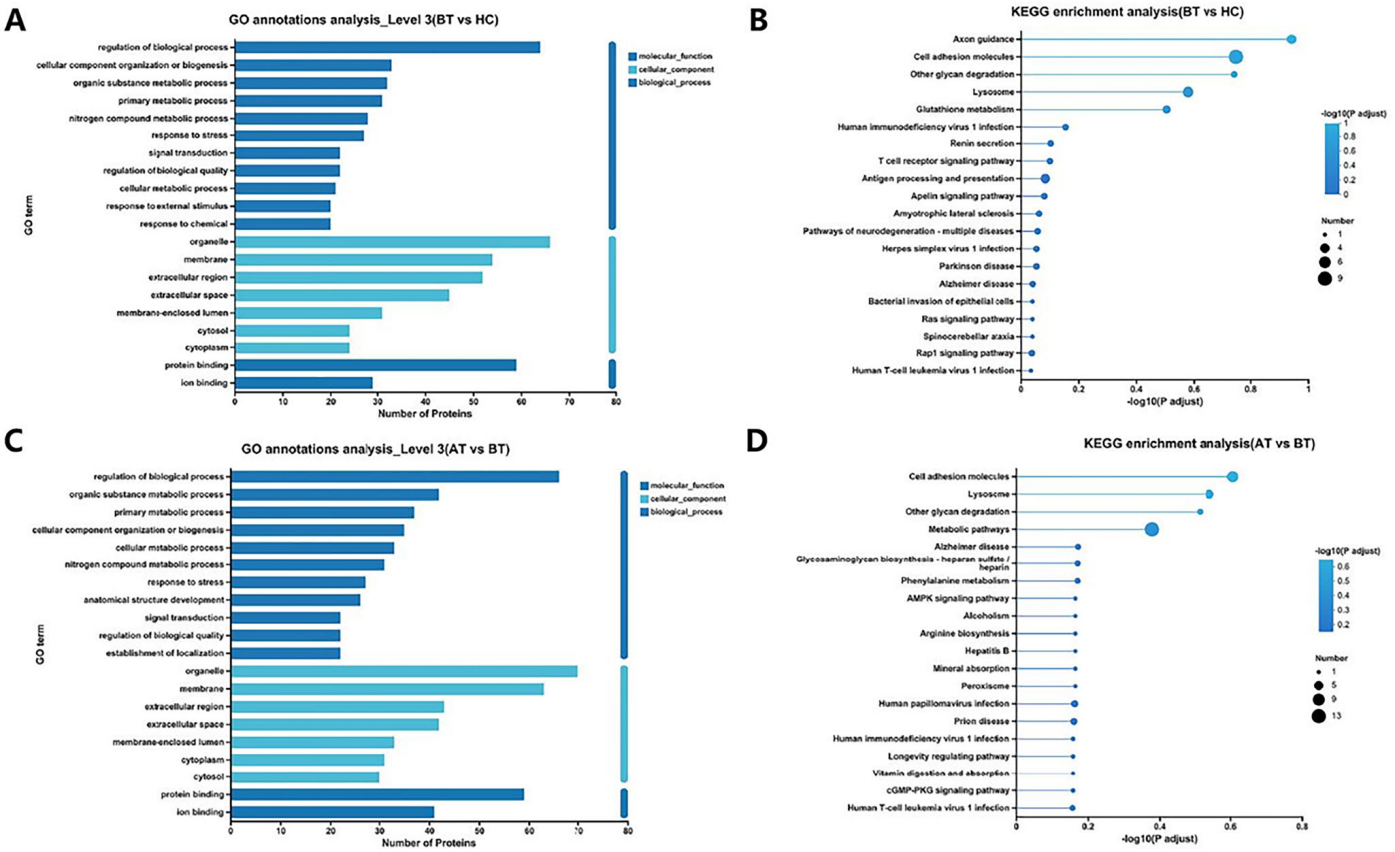
GO and KEGG analysis of DEPs. (A) GO functional annotation analysis of the disease‐associated DEPs. (B) KEGG functional enrichment analysis of the disease‐associated DEPs. (C) GO functional annotation analysis of the acupuncture treatment‐associated DEPs. (D) KEGG functional enrichment analysis of the acupuncture treatment‐associated DEPs.

Figure 3A displays the GO functional annotation outcomes for DEPs between ACI patients and healthy controls. Regarding biological processes, the top three enriched terms associated with ACI proteins were cellular process, biological regulation, and response to stimulus. Cellular component analysis showed that the most highly enriched categories were cellular anatomical entity and protein‐containing complex. Molecular function analysis revealed that the predominant functions were binding, catalytic activity, and molecular function regulator activity.

To further examine significantly enriched pathways in DEPs when comparing ACI patients with healthy controls, the DEPs were aligned to the KEGG database and enrichment scores were derived. As shown in Figure 3B, KEGG pathway enrichment analysis indicated that disease‐related DEPs were mainly enriched in several pathways, notably cell adhesion molecules, axon guidance, and lysosome.

### 2.5 GO and KEGG Analysis of Acupuncture Treatment‐Associated DEPs

Figure 3C presents GO annotation of the 342 DEPs in ACI patients after acupuncture treatment. The top three enriched biological processes were cellular process, biological regulation, and metabolic process. Cellular component analysis showed cellular anatomical entity and protein‐containing complex as the most enriched. Molecular function analysis identified binding, catalytic activity, and molecular function regulator activity as the predominant functions.

Figure 3D shows KEGG enrichment results for these DEPs. Treatment‐associated DEPs were mainly enriched in metabolic pathways, cell adhesion molecules, and lysosome.

### 2.6 Protein–Protein Interaction (PPI) Network Analysis

Using the STRING PPI database, we performed PPI network analysis on two distinct sets of DEPs: 340 disease‐associated DEPs (BT vs. HC) and 342 treatment‐responsive DEPs (AT vs. BT). The analysis revealed 55 interacting protein pairs exhibiting aberrant expression in the serum of ACI patients, which may be associated with abnormal pathophysiological processes, and 36 interacting protein pairs potentially linked to the therapeutic mechanism of acupuncture in treating ACI (Table 3; Figure S2). Protein interactions were extracted using a confidence score higher than 0.7 (high confidence), and two shared interaction pairs were identified across both protein subsets: FUCA2‐FUCA1 and SPP1‐ACAN. Detailed results are presented in Table 3 and Figure S3).

**TABLE 3:** Differential proteins with highly interactive relationships (disease‐related).

BT vs. HC			AT vs. BT		
Protein name (A)	Protein name (B)	Combined score	Protein name (A)	Protein name (B)	Combined score
C4BPA	C4A	0.998	VCAN	ACAN	0.988
CRP	CFHR4	0.985	LTF	CP	0.971
SAA1	SAA2	0.975	CP	SERPINA1	0.944
CP	HP	0.947	COL3A1	GP6	0.936
**FUCA2**	**FUCA1**	0.928	**FUCA2**	**FUCA1**	0.928
CRP	HP	0.902	TGM3	SBSN	0.833
CRP	ACE	0.878	LTF	SLPI	0.811
CRP	FCN1	0.866	LTF	SPP1	0.772
CRP	SAA1	0.854	**SPP1**	**ACAN**	0.736
HP	C4A	0.817	VCAN	COL3A1	0.722
CRP	SAA2	0.811			
CRP	C4A	0.787			
IHH	ACAN	0.761			
CRP	C4BPA	0.76			
HP	SAA2	0.751			
FCN1	C4A	0.744			
HP	SAA1	0.738			
**SPP1**	**ACAN**	0.736			
CRP	PLA2G7	0.715			
CCN2	ACAN	0.712			

*Note*: The bolded proteins denote the differentially expressed proteins shared by both comparison groups (BT vs. HC and AT vs. BT), namely SPP1, ACAN, FUCA2, and FUCA1.

### 2.7 Verification of Proteins Identified in Serum Samples

**FIGURE 4:**
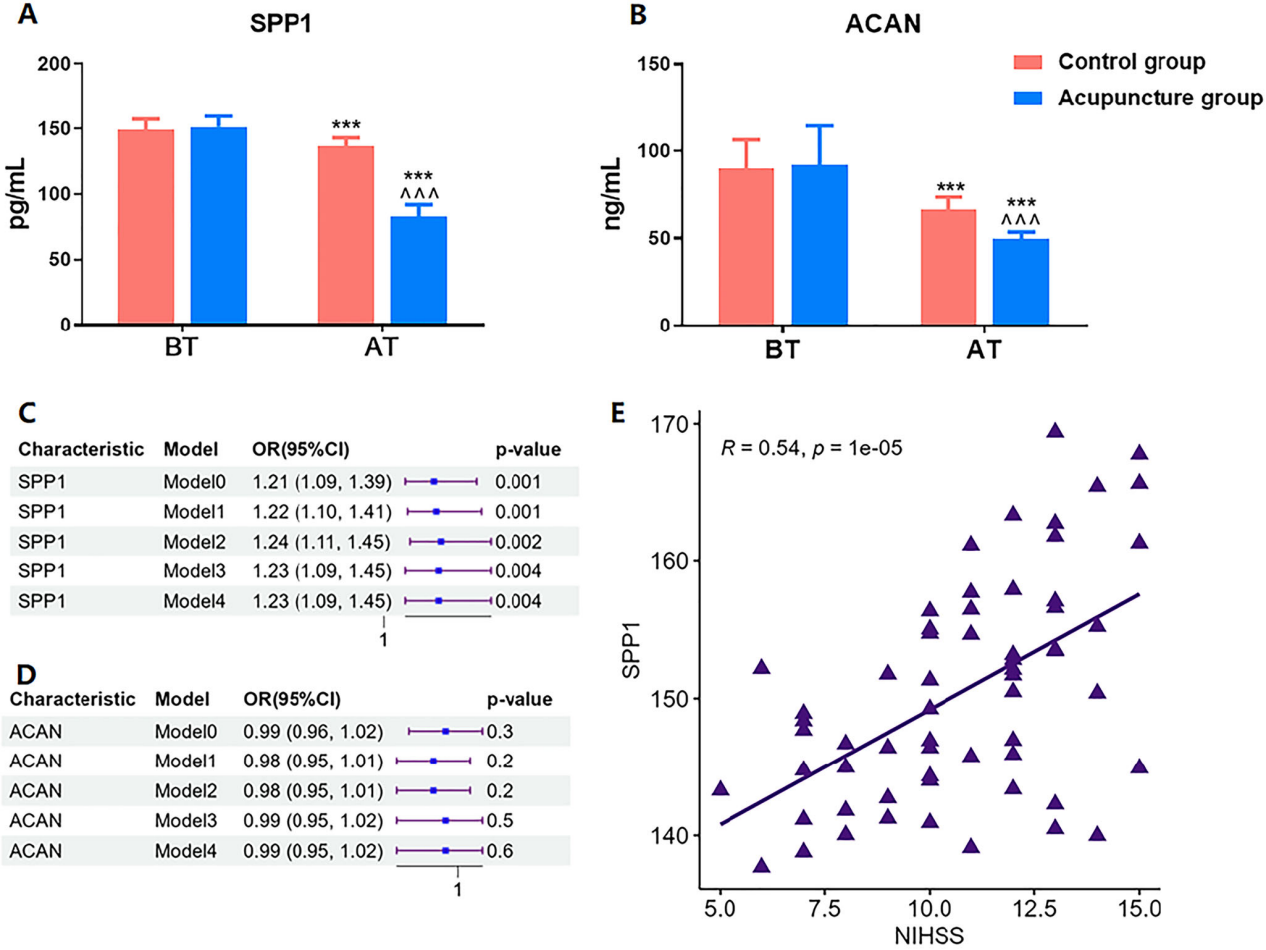
SPP1 was identified as the target protein in the serum of ACI patients through the ELISA validation and correlation analysis. (A) The expression of SPP1 in the serum of ACI patients by ELISA. (B) The expression of ACAN in the serum of ACI patients by ELISA. (C) The association between baseline expression levels of SPP1 and severe stroke (NIHSS > 10) evaluated using univariate and multivariable logistic regression analysis. Model 0: unadjusted; Model 1: adjusted for age and sex; Model 2: additionally adjusted for drinking and smoking status; Model 3: additionally adjusted for diabetes, CAD, and hypertension; Model 4: fully adjusted, additionally including previous stroke. (D) The association between baseline expression levels of ACAN and severe stroke (NIHSS > 10) evaluated using the same sequential adjustment models as in (C). (E) Correlation analysis between the expression of SPP1 and NIHSS was performed using the Pearson correlation coefficient.

We selected SPP1 and aggrecan (ACAN) as target proteins and validated the proteomic results in an independent validation cohort comprising 60 patients with ACI (30 per group: acupuncture vs. control). Utilizing enzyme‐linked immunosorbent assay (ELISA), we quantified SPP1 and ACAN expression pre‐ and posttreatment in both groups. Statistical analysis demonstrated a significant decrease in the expression levels of both proteins posttreatment compared to baseline in all patients (*p* < 0.001). Crucially, posttreatment levels in the acupuncture group were significantly lower than those in the control group (*p* < 0.001). These ELISA findings are concordant with the initial proteomic analysis (Figure 4A,B).

The association between the expression levels of SPP1/ACAN and severe stroke (defined as NIHSS > 10) was further evaluated using univariate and multivariable logistic regression analysis. The results shown that the higher expression of SPP1 was consistently associated with a significantly increased risk of severe stroke across all models. Specifically, in the unadjusted model (Model 0), the OR was 1.21 (95% CI: 1.09–1.39, *p* = 0.001) per 1‐unit increase in baseline SPP1 expression. This association remained stable and significant through successive adjustments: Model 1 (adjusted for age and sex; OR: 1.22, 95% CI: 1.10–1.41, *p* = 0.001), Model 2 (additionally adjusted for alcohol consumption and smoking status; OR: 1.24, 95% CI: 1.11–1.45, *p* = 0.002), Model 3 (additionally adjusted for diabetes, CAD, and hypertension; OR: 1.23, 95% CI: 1.09–1.45, *p* = 0.004), and fully adjusted Model 4 (additionally adjusted for previous stroke; OR: 1.23, 95% CI: 1.09–1.45, *p* = 0.004), indicating the robustness of SPP1 as independently associated with severe stroke. In contrast, ACAN expression showed no significant association with severe stroke in any model. The ORs per 1‐unit increase in baseline ACAN expression were consistently close to 1.0 across all tiers of adjustment, from Model 0 (OR: 0.99, 95% CI: 0.96–1.02, *p* = 0.3) to the fully adjusted Model 4 (OR: 0.99, 95% CI: 0.95–1.02, *p* = 0.6), confirming the lack of an independent relationship. Finally, a moderate positive correlation was found between SPP1 expression and NIHSS (*R* = 0.54, *p* < 0.001). This moderate correlation suggests that higher expression levels of the biomarker are associated with increased neurological deficit severity, as quantified by the NIHSS.

### 2.8 Acupuncture Intervention Reduces Acute Ischemic Brain Injury

**FIGURE 5:**
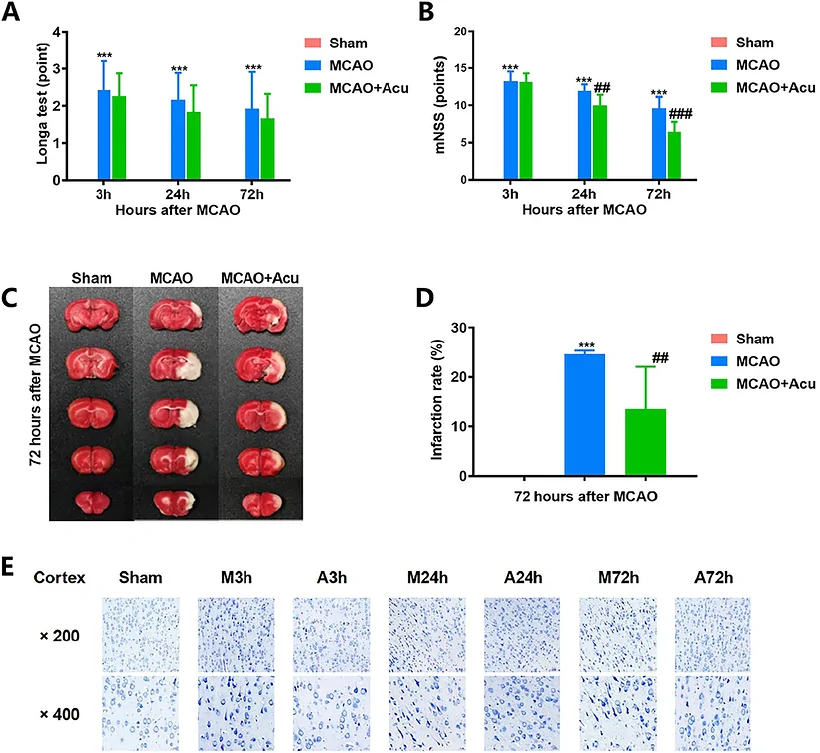
Acupuncture enhanced neurological outcomes in MCAO rat. (A) Neurological functional change after MCAO in rats as shown by Longa test (*n* = 12). (B) Neurological functional change after MCAO in rats as shown by modified neurological severity score (*n* = 12). (C) TTC assay was conducted to assess the cerebral infarct volume at 72 h after MCAO (*n* = 6). (D) Cerebral infarct volume rate was calculated at 72 h after MCAO (*n* = 6). (E) Representative photomicrographs of Nissl staining results of each group (original magnification, ×200 and ×400; *n* = 3). *p < 0.05 vs. Sham, #*p* < 0.05 vs. MCAO. Bars represent mean ± SD.

We employed an MCAO rat model to assess the neuroprotective effects of acupuncture against ACI. Neurological functions were first assessed by performing Longa test and mNSS after MCAO surgery. MCAO rats had more severe neurological deficits than the Sham rats, and the intervention of acupuncture rats were less impaired in the Longa test and mNSS score at 3, 24, and 72 h after MCAO (Figure 5A,B). Compared with rats of the MCAO group, rats treated with acupuncture exhibited significantly reduced infarct volume at 72 h after MCAO (Figure 5C,D). Nissl staining revealed that most cells in the ischemic cortex of the MCAO group exhibited cellular shrinkage, widened intercellular spaces, and intense staining, indicating ischemia‐induced injury. Contrastingly, acupuncture intervention attenuated these pathological changes at 3, 24, and 72 h after MCAO (Figure 5E).

### 2.9 Acupuncture Intervention Ameliorates MCAO‐Induced Neuroinflammation in Rats

**FIGURE 6:**
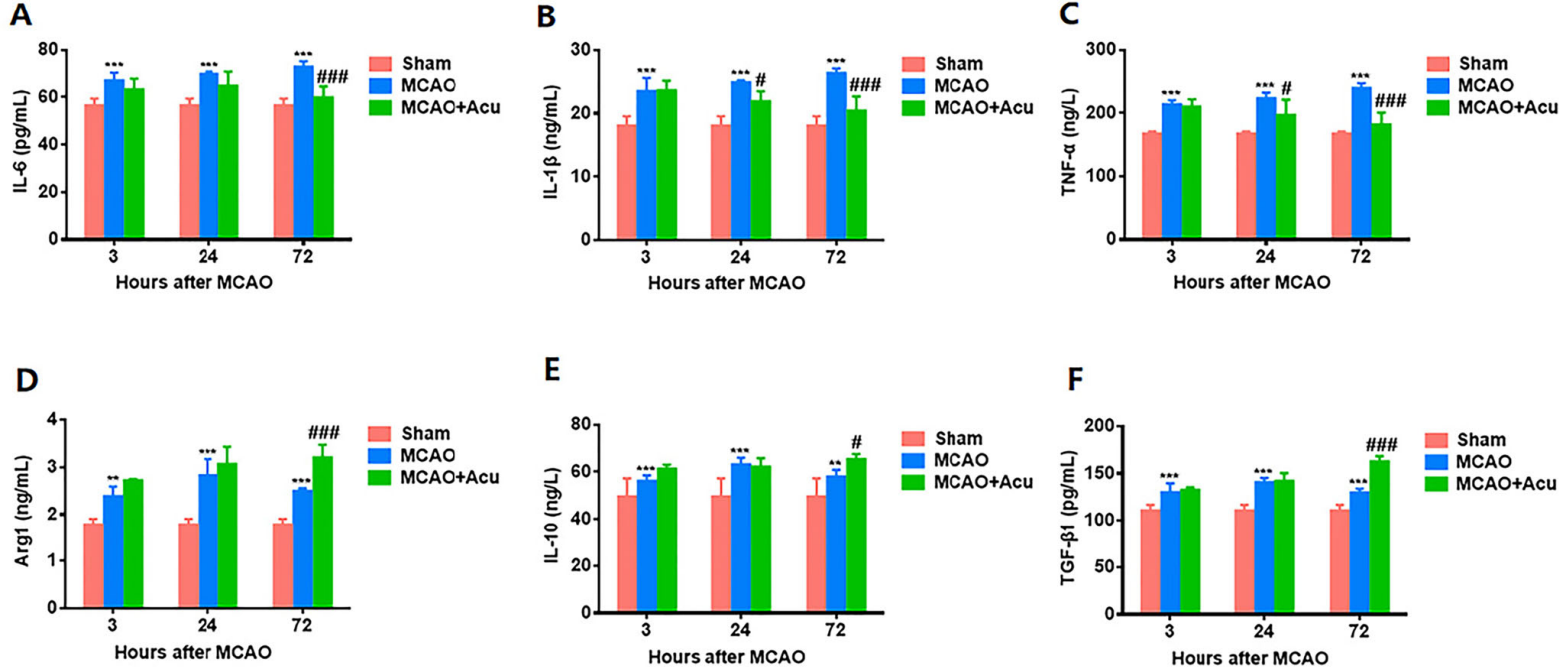
Expression of acupuncture on inflammatory cytokines were measured by ELISA on brain tissue at 3, 24, and 72 h after MCAO. (A–C) Expression of M1 phenotype factors (IL‐6, IL‐1β, TNF‐α), (D–F) Expression of M2 phenotype factors (Arg‐1, IL‐10, TGF‐β1). *p < 0.05 vs. Sham, ^#^ *p* < 0.05 vs. MCAO. Bars represent mean ± SD (*n* = 3).

We profiled the levels of six cytokines, using the ELISA, to explore the inflammatory status of brain tissue at 3, 24, and 72 h after MCAO. Notably, three pro‐inflammatory cytokines (including IL‐6, IL‐1β, and TNF‐α) and three anti‐inflammatory cytokines (including Arg‐1, IL‐10, and TGF‐β1), which were significantly elevated in the MCAO group compared with sham‐operated rats. Acupuncture significantly reduced the levels of pro‐inflammatory cytokines and elevated the level of anti‐inflammatory cytokine compared with MCAO group (Figure 6).

**FIGURE 7:**
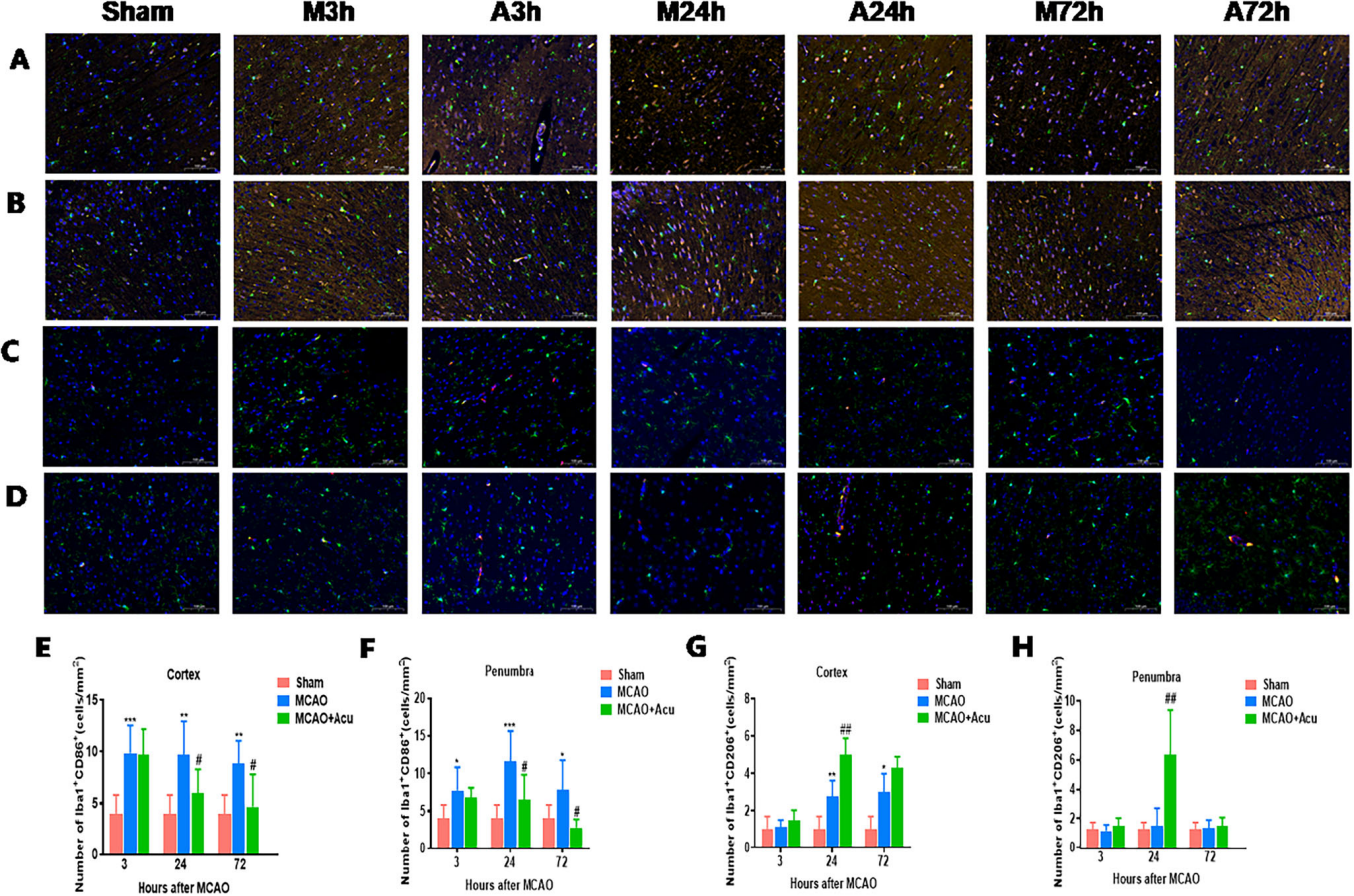
Representative images of immunofluorescence analysis for microglia polarization. Double immunofluorescent staining for CD86 (orange; pro‐inflammatory microglia marker) and Iba1 (green) in cortex (A) and penumbra (B). Double immunofluorescent staining for CD206 (red; anti‐inflammatory) and Iba1 (green) in cortex (C) and penumbra (D). Quantitative analysis of pro‐inflammatory microglia in cortex (E) and penumbra (F), and anti‐inflammatory microglia in cortex (G) and penumbra (H). *p < 0.05 vs. Sham, ^#^ *p* < 0.05 vs. MCAO. Bars represent mean ± SD (*n* = 3–6, scale bar: 100 µm).

The results shown in Figure 6 prompted us to explore whether acupuncture could promote microglial M1/M2 phenotypic polarization. Figure 7A–H illustrates that double staining for CD86, an M1 phenotype marker, and Iba1 revealed a significant decrease in CD86+/Iba1+ cells in the cortex and penumbra at 24 and 72 h after MCAO with acupuncture treatment. Conversely, images showing double staining of CD206, an M2 phenotype marker, with Iba1 indicated a significant increase of CD206 + /Iba1 + cells in the cortex and penumbra of MCAO rats treated with acupuncture at 24 h after MCAO.

### 2.10 Microglia‐Vessel Accumulation in MCAO Rats and the Effect of Acupuncture Intervention

**FIGURE 8:**
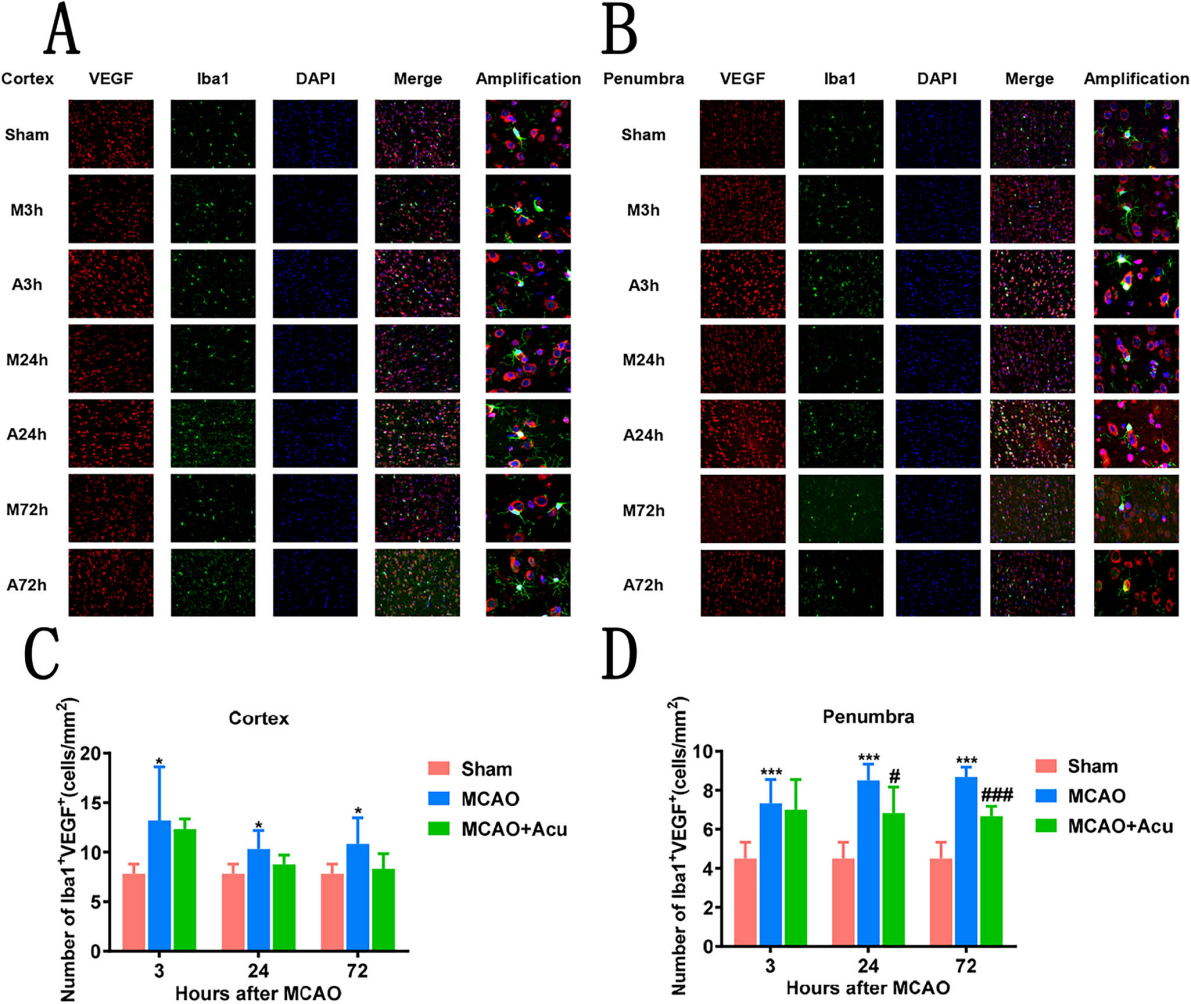
Co‐localization of microglia with VEGF‐positive vascular structures in cortex and penumbra after MCAO. Double immunofluorescent staining for VEGF (red; VEGF‐positive signals) and Iba1 (green, microglia marker) in cortex (A) and penumbra (B). Quantitative analysis of cell counts showed that acupuncture significantly reduced the number of VEGF^+^/Iba1^+^ cells in the cortex (C) and penumbra (D) of MCAO rats. *p < 0.05 vs. Sham, ^#^ *p* < 0.05 vs. MCAO. Bars represent mean ± SD (*n* = 3–6, scale bar: 100 µm).

Microglia are the primary immune cells activated in hypoxia and maintain bidirectional interactions with VECs (Dudvarski Stankovic et al. 2016). We therefore examined microglial responses in the MCAO rat model of ACI. Immunohistochemical staining for Iba1 (microglia) and VEGF was performed. Because VEGF is robustly upregulated in endothelial cells and perivascular tissues after ischemia, VEGF immunoreactivity was utilized to delineate cerebral vessels, with the caveat that it is not a constitutive endothelial structural marker. In the cortex and penumbra, an increased number of Iba1^+^ microglia were found closely associated with VEGF‐positive vascular structures at 3, 24, and 72 h after MCAO. Acupuncture intervention reduced this microglial apposition to VEGF^+^ vessels, particularly in the penumbra at 24 and 72 h post‐MCAO (Figure 8).

### 2.11 Distribution of SPP1 and the Effect of Acupuncture Intervention

**FIGURE 9:**
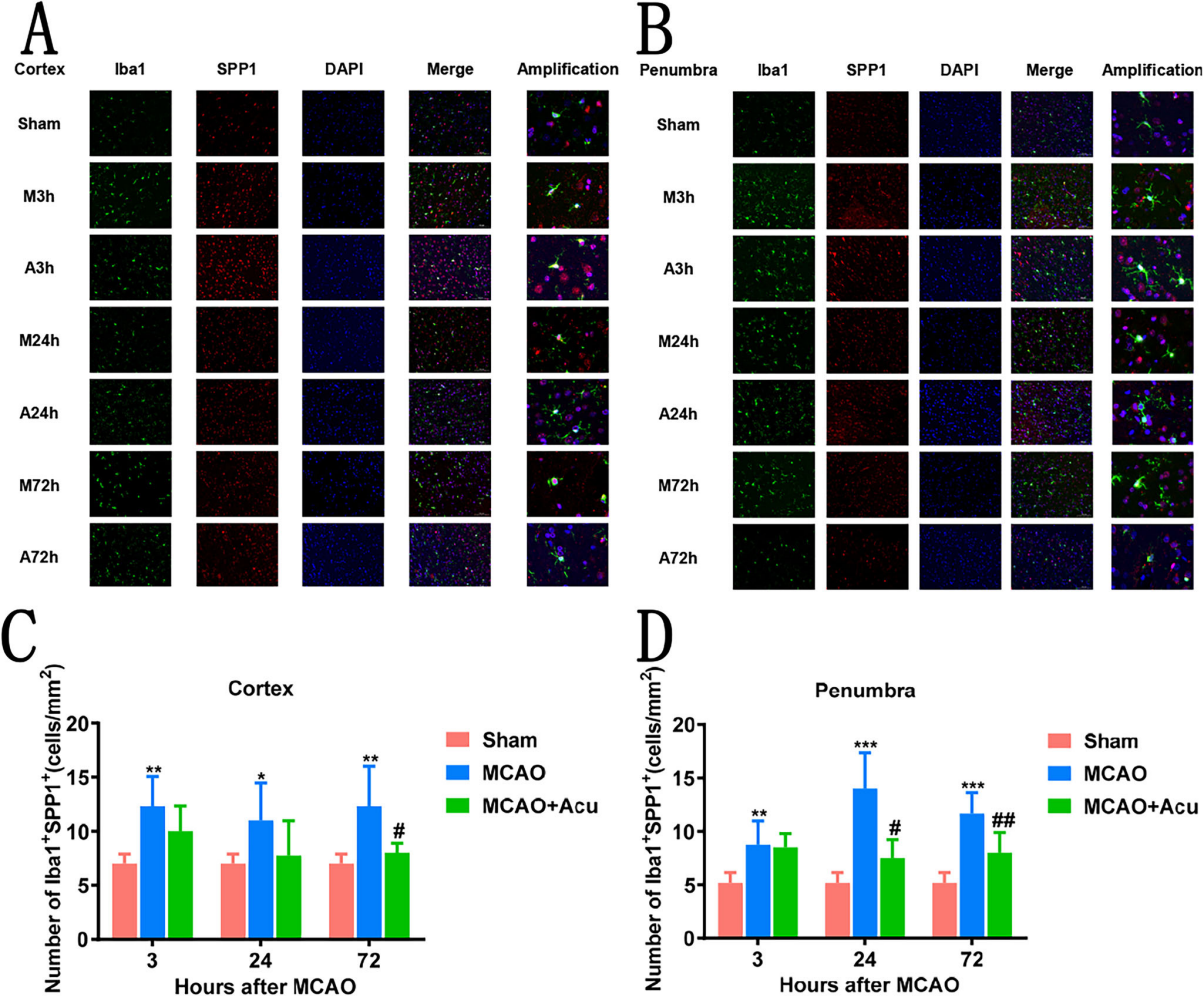
Co‐localization of SPP1 with microglia after MCAO. Double immunofluorescent staining for SPP1 (red) and Iba1 (green) in cortex (A) and penumbra (B). Quantitative analysis of cell counts showed that acupuncture significantly reduced the number of SPP1 + /Iba1 + cells in the cortex (C) and penumbra (D) of MCAO rats. *p < 0.05 vs. Sham, ^#^ *p* < 0.05 vs. MCAO. Bars represent mean ± SD (*n* = 3–6, scale bar: 100 µm).

**FIGURE 10:**
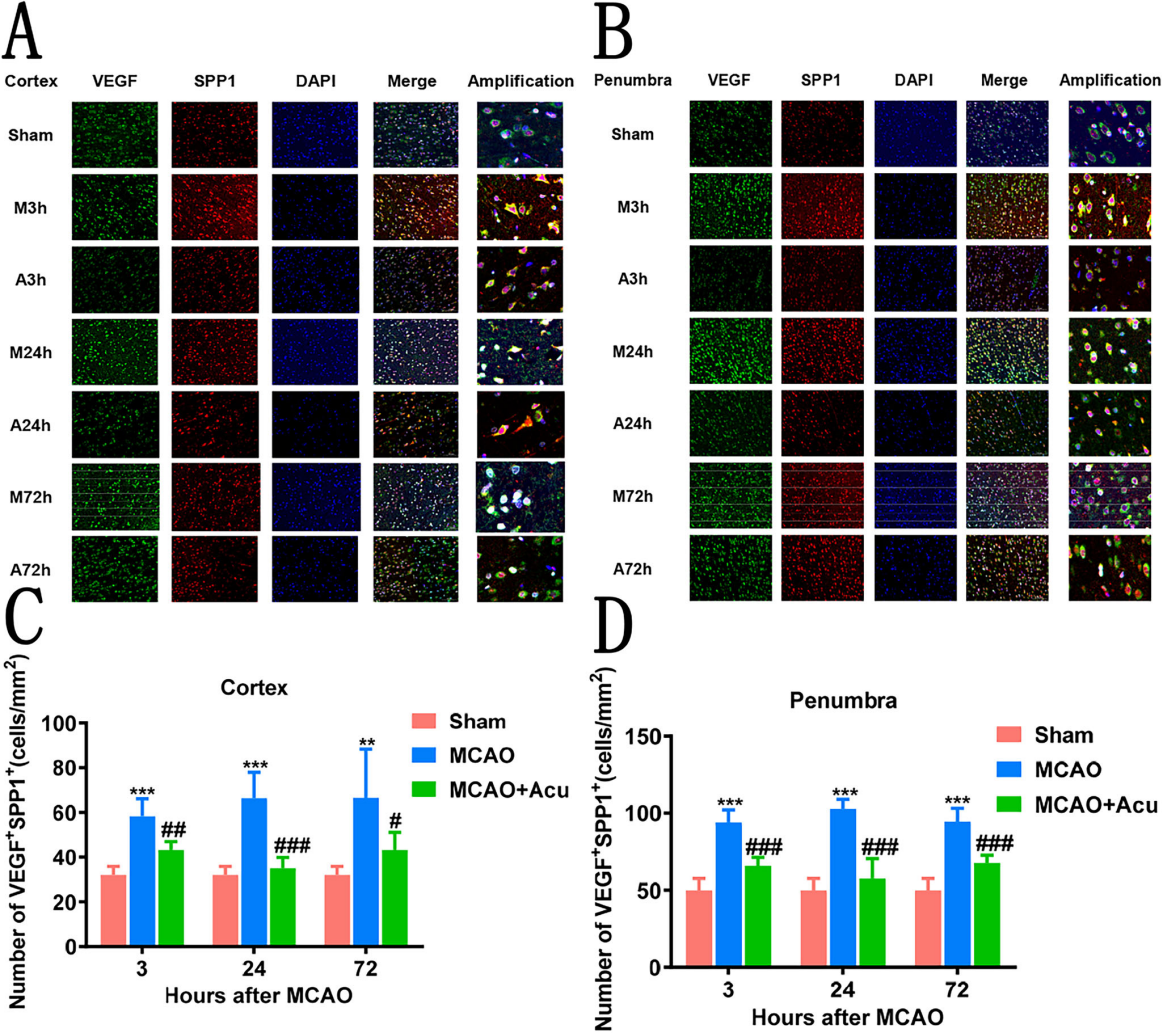
Co‐localization of SPP1 with VEGF‐positive vascular structures after MCAO. Double immunofluorescent staining for SPP1 (red) and VEGF‐positive signals (green) in cortex (A) and penumbra (B). Quantitative analysis of cell counts showed that acupuncture significantly reduced the number of SPP1 + /VEGF + cells in the cortex (C) and penumbra (D) of MCAO rats. *p < 0.05 vs. Sham, ^#^ *p* < 0.05 vs. MCAO. Bars represent mean ± SD (*n* = 3–6, scale bar: 100 µm).

SPP1, also known as osteopontin, is an extracellular matrix constituent of the central nervous system, and a proinflammatory cytokine (Yu et al. 2017). To explore its distribution, we performed double immunostaining for SPP1 together with Iba1 (microglia) and VEGF. The results showed a significant increase in SPP1^+^/Iba1^+^ cells in the cortex and penumbra after MCAO, which was reduced by acupuncture treatment at 3, 24, and 72 h (most notably in the cortex at 72 h and in the penumbra at 24 and 72 h) (Figure 9). Furthermore, SPP1/VEGF double staining revealed a marked rise in SPP1^+^/VEGF^+^ signals in both regions, and this elevation was also attenuated by acupuncture at all three time points (Figure 10).

### 2.12 Acupuncture Downregulated SPP1 Expression via CD200/CD200R Signaling Pathway in MCAO Rats

**FIGURE 11:**
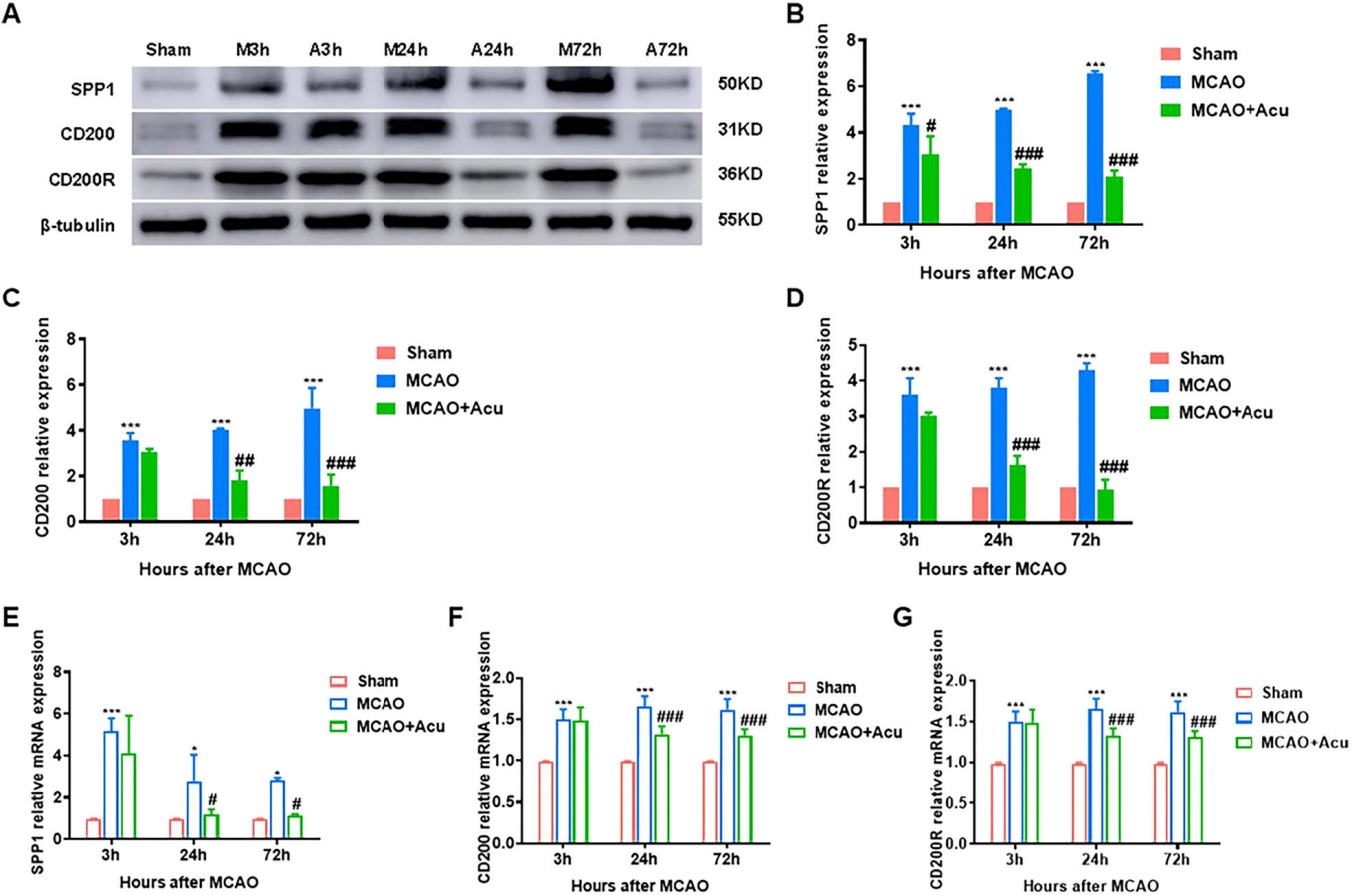
Acupuncture downregulated SPP1 expression via CD200/CD200R signaling pathway after MCAO. Representative bands of the western blot (A) and quantitative analysis of SPP1 (B), CD200 (C), and CD200R (D) normalized to β‐tubulin. Transcript profile analysis for genes of SPP1 (E), CD200 (F), and CD200R (G) conducted by RT‐qPCR. *p < 0.05 vs. Sham, ^#^ *p* < 0.05 vs. MCAO. Bars represent mean ± SD (*n* = 3).

CD200 is extensively expressed on neurons and other cells, mediating inhibitory signals through its receptor, CD200R, found on myeloid lineage cells like macrophages and microglia (Hoek et al. 2000; Sun et al. 2020). To explore whether the anti‐inflammation effects of acupuncture are associated with the downregulation of the SPP1‐mediated CD200/CD200R pathway, the expression level of the protein and mRNA were further determined by WB and RT‐qPCR. As detected by western blotting, there was a notable rise in the protein levels of SPP1, CD200, and CD200R at 3, 24, and 72 h. In contrast, the expression of SPP1, CD200, and CD200R was reduced markedly after acupuncture intervention. The RT‐qPCR results were consistent with the WB findings (Figure 11).

## 3 Discussion

Ischemic stroke is the most widespread subtype, making stroke a primary cause of death and disability around the world (GBD 2019 Stroke Collaborators 2021). The numbers of studies have been reported that neuroinflammation plays a significant role in the pathophysiological process of ACI, potentially aggravating brain injury and hindering neuro‐repair (Liu et al. 2021; Tang et al. 2022; Qian et al. 2024; Liao et al. 2025). Acupuncture, as a kind of traditional medicine, reveals a promising anti‐inflammatory effect in cerebral ischemic, but the mechanism remains unclear (Ren et al. 2024; S. Chen et al. 2024). In this study, we identified the contribution of an extracellular matrix protein, SPP1, as a pro‐inflammatory cytokine involved in the regulation of neuroinflammatory events by acupuncture following ACI.

SPP1 is a highly acidic protein with a capacity of 45–75 kDa, which participates in multiple cellular pathways by using (1) discrete functional domains, (2) as substrates for specific proteases, (3) posttranslational modifications, and (4) the activity of splicing variants. A recent study, through the integrated analysis of transcriptome data from mice, rats after MCAO and primary cell transcriptional datasets, found that SPP1 played a negative regulation of inflammation and was upregulated in both transient and permanent MCAO (Lv et al. 2023). Furthermore, another study has shown that anti‐SPP1 antibody therapy may enhance currently approved reperfusion strategies for ACI by mitigating the detrimental effects associated with ischemia‐induced blood‐brain barrier disruption (Spitzer et al. 2022). Our results confirm that the levels of SPP1 were increased in serum of patients with ACI and in brain tissue of MCAO rats, which were in consistent with the above findings. And the levels of SPP1 were decreased with the treatment of acupuncture paralleled by alleviated neurological deficits, decreased infarction volume, reduced neuronal damage, and suppressed neuroinflammation.

Immune cells around the cerebral vessels share numerous similarities, but their varied locations hint at differing functions. Despite recent efforts to differentiate these cell populations, such as capillary‐associated microglia (Bisht et al. 2021), perivascular microglia (Hickey and Kimura 1988), or VAMs (Koizumi et al. 2019), their respective roles and dynamics in cerebrovascular disease pathogenesis are still not clearly defined. In this study, we found that acupuncture not only inhibited the recruitment of microglia to the infarcted vessels, but also promoted the polarization of microglia toward the M2 phenotype, thereby attenuating neuroinflammation and alleviating neuronal during MCAO. SPP1 is a multifunctional protein which has been performed with cells in the peripheral immune system, but the functions of SPP1 in the CNS have yet to be largely explored. Microglia and astrocyte play critical roles in CNS inflammation, which are most intensively studied cell types of CNS‐resident cells regarding SPP1 (Lin et al. 2023). Interestingly, at the early phase of MCAO, co‐expression of SPP1 and microglia, SPP1 and VEGF‐positive signals increased, of which was efficiently reduced by acupuncture treatment.

The above results indicate that after cerebral infarction occurs, microglia rapidly aggregate in the infarcted tissue and undergo phenotypic transformation, leading to neurological deficits and severe neuroinflammation. Next, we further investigated the potential mechanism by which SPP1 mediates the anti‐inflammatory effects of acupuncture on neuroinflammation. Recently, CD200/CD200R signaling pathway has been increasingly investigated, and previous studies have shown that CD200/CD200R signaling pathway plays an important role in neuroinflammation after stroke (Sun et al. 2020; Ritzel et al. 2019). Given these research facts, we hypothesized that acupuncture may regulate microglia‐vascular crosstalk by reducing the expression of SPP1, thereby improving the immunological niches. To further verify this hypothesis, we used Western blot and real‐time quantitative PCR method to detected the expression of proteins and genes related to the CD200/CD200R signaling pathways. Our results showed an overall decrease in the detection of the proteins assayed after acupuncture intervention. The RT‐qPCR assay results were consistent with the Western blot results.

There are limitations in current studies that should be considered in future research. First, the acupuncture method employed in clinical application and animal experiment are different, and accurate dose conversion between animals and human is challenging. Second, the specific origin of SPP1 remain to be determined and need more study. Third, our discovery that acupuncture exerts anti‐inflammatory effects by inhibiting the expression of SPP1 in regulating the CD200/CD200R signaling pathway requires further in vivo and in vitro experiments for verification. Future research aimed at addressing these limitations will be critical for advancing our mechanistic understanding and enhancing the translational applicability of findings.

## 4 Conclusions

In summary, our research findings suggest that acupuncture may modulate neuroinflammation through the downregulation of SPP1 protein expression. By reducing SPP1 expression in MCAO models, acupuncture may influence the crosstalk between microglia and VECs, thereby improving the immunological niches and exerting anti‐inflammatory effects. This process may be associated with the CD200/CD200R signaling pathway. These results elucidate the anti‐inflammatory mechanisms underlying SPP1‐related acupuncture treatment for cerebral infarction and deserve future investigations of its potential clinical implications.

## 5 Materials and Methods

### 5.1 Study Design and Population

Peripheral blood samples utilized in this work were derived from a multicenter, sham‐controlled, randomized clinical trial that applied blinding for both patients and outcome assessors. The trial was conducted at four tertiary hospitals across China and received approval and oversight from the Institutional Review Board of the First Teaching Hospital of Tianjin University of Traditional Chinese Medicine. The protocol (registered on http://www.chictr.org.cn, identifier ChiCTR2300079204, on December 27, 2023) and the primary clinical outcomes have been reported previously (Cao et al. 2025; Cao et al. 2024). Briefly, 80 participants were assigned to two independent cohorts: Cohort 1 comprised 10 ACI patients and 10 healthy controls, while Cohort 2 included 60 ACI patients who were allocated to an acupuncture group or a control group. Blood was drawn from the ACI patients before treatment (BT) and after treatment (AT).

### 5.2 Clinical Intervention and Proteomic Analysis

All patients enrolled in two cohorts received standard according to the Chinese guidelines for the diagnosis and treatment of acute ischemic stroke 2018 (Neurology and Society 2018). In addition, the patients allocated to the acupuncture group underwent 12 sessions of acupuncture, delivered six times weekly over 2 weeks, with each session lasting 30 min. The blood samples collection and classification, proteomic analysis, DEPs selection and validation, and statistical analysis followed a previously published protocol (Cao et al. 2024).

### 5.3 ELISA

Peripheral blood from patients and brain tissues from rats were collected and kept at 4°C. Plasma SPP1 and ACAN concentrations were measured using human ELISA kits (mlbio, Shanghai) according to the manufacturer's instructions. Brain tissues were homogenized in RIPA buffer, and protein concentration was quantified with a BCA kit (Beyotime, Nanjing, China). Arg1, IL‐10, TGF‐β1, IL‐1β, IL‐6, and TNF‐α levels were then determined by ELISA kits (FANKEW, Shanghai, China) following the suppliers’ protocols.

### 5.4 Animals, MCAO Procedure, and Acupuncture Intervention

Adult male Sprague‐Dawley rats (260–280 g, 8–10 weeks of age) were purchased from Beijing Huafukang Bioscience Co., Ltd. (Beijing, China), and housed at 22 ± 2°C, 60 ± 10% humidity, under a 12/12 h light/dark cycle with ad libitum food and water for ≥1 week before experiments. All animal procedures were approved by the Animal Ethics Committee of Tianjin University of Traditional Chinese Medicine (TCM‐LAEC2024020h1555) and complied with the Guide for the Care and Use of Laboratory Animals (National Academy of Sciences, USA).

A permanent MCAO model was performed as described previously (Du et al. 2011). Briefly, rats were anesthetized with 5% isoflurane and maintained at 3% during surgery. Following a midline cervical incision, the right common carotid artery (CCA) was exposed. The external carotid artery (ECA) was dissected from the CCA and internal carotid artery (ICA), then ligated at its origin. Microsurgical clips were placed on the proximal CCA and ICA origin. The CCA was punctured between the clips with a 2 mL needle, and a blunted nylon suture was inserted. After removing the ICA clip, the suture was advanced into the ICA until mild resistance, then the suture and CCA puncture site were ligated, the remaining clip removed, and the incision closed. Sham‐operated rats underwent the same procedures without MCA occlusion. Body temperature was kept at 37°C–37.5°C using a heating pad until recovery.

To avoid restraint stress, rats were anesthetized. Acupuncture group rats received stimulation at Shuigou (GV26) beginning 3 h post‐MCAO, using 0.3 mm × 25 mm needles inserted 2 mm deep for 20 min, once daily for three consecutive days.

### 5.5 Neurobehavioral Assessment

At 3, 24, and 72 h post MCAO, an investigator blinded to the group allocation assessed neurological deficits using the Zea Longa (0–4) score (Longa et al. 1989) and the modified neurological severity score (mNSS, 0–18) (Morris et al. 2010); higher scores indicate more severe deficits.

### 5.6 TTC Staining

Brains were harvested 72 h after MCAO, stored at −20°C for 2 h, and then sliced into five 2‐mm coronal sections. The sections were stained with 2% TTC (Solarbio, China) at 37°C for 30 min, fixed in 4% paraformaldehyde at 4°C for 4 h, and photographed. Total infarct volume was quantified using ImageJ.

### 5.7 Nissl Staining

At 3, 24, and 72 h after MCAO, brains were fixed in 4% paraformaldehyde, embedded in paraffin, and sliced into 10‐µm coronal sections. Sections were stained with Nissl solution (G1430, Solarbio, Beijing, China) for 30 min at 37°C, dehydrated through a graded ethanol series (70%, 80%, 90%, and twice 100% ethanol, 30 s each), air‐dried, and mounted with neutral resin.

### 5.8 Immunofluorescent Staining

Immunofluorescence was performed on 10% formalin‐fixed brain sections. After blocking with BSA, sections were incubated overnight at 4°C with primary antibodies: anti‐Iba1 (1:500), anti‐CD86 (1:200), anti‐CD206 (1:500) (HUABIO, ZheJiang, China), anti‐VEGF (1:200), and anti‐SPP1 (1:200) (BIOSS, Beijing, China). Following washes, sections were incubated with secondary antibodies for 1 h at room temperature, washed, and imaged with an Olympus fluorescence microscope. ImageJ was used for analysis.

### 5.9 Western Blot Assays

Total proteins from brain tissues were extracted at 3, 24, and 72 h post‐MCAO using RIPA buffer (Beyotime, Nanjing, China) containing 1 mM PMSF and protease inhibitor (Solarbio, Beijing, China). Protein concentration was determined with a BCA kit (Beyotime, Nanjing, China). Equal protein amounts were resolved by SDS‐PAGE and transferred to PVDF membranes (Millipore, MA, USA). Membranes were blocked with 5% skim milk for 2 h at room temperature, then incubated overnight at 4°C with primary antibodies: SPP1(1:1000, BIOSS), CD200(1:1000, BOSTER), and CD200R (1:1000, GeneTex). After incubation with HRP‐conjugated secondary antibodies for 120 min, signals were developed with ECL reagents (Millipore, MA, USA), and analyzed with ImageJ.

### 5.10 Real‐Time Quantitative RT‐PCR (RT‐qPCR)

Total RNA was isolated from brain tissues at 3, 24, and 72 h after MCAO using TRIzol reagent (ThermoFisher Scientific, Waltham, MA USA) following the manufacturer's protocol. Equal amounts of total RNA were reverse‐transcribed with the HiScript first Strand cDNA Synthesis Kit (Accurate biology, Hunan, China). Quantitative real‐time PCR was then performed on an ABI7000 system (Applied Biosystems, Inc., Carlsbad, CA, USA) with SYBR Green Master Mix (Accurate biology, Hunan, China). mRNA expression levels were normalized to β‐tubulin, and relative expression was calculated by the 2^−ΔΔCt^ method. Primer sequences are listed in Table 4.

**TABLE 4:** Primer sequences.

Gene	Forward primer (5′–3′)	Reverse primer (5′–3′)
*SPP1*	CGATGATGACGACGACGATGA	GTGCTGGCAGTGAAGGACTC
*CD200*	TTCTGCCATCTGTCCACCTACA	GGTCACCACTTCCACTTGAGC
*CD200R*	ACAAAGCAGACACAAGGGAGAC	AAGGTCAGGAGCGAGGTCAG
*β‐tubulin*	GACACGGACAGGATTGACAGAT	TGCCAGAGTCTCGTTCGTTATC

### 5.11 Statistical Analysis

Data are presented as mean ± standard deviation and performed using GraphPad Prism Software 6 (La Jolla, CA, USA). Multiple‐group comparisons were performed by one‐way or two‐way ANOVA, correlations were assessed by Pearson correlation coefficient and multivariable logistic regression, and *p* < 0.05 was considered statistically significant. DEPs were selected using R with a stringent |log_2_FC| ≥ 2 and unadjusted *p* < 0.05, while the Benjamini–Hochberg correction was applied in the KEGG pathway enrichment analysis performed in Python.

## Author Contributions

Jiang‐Peng Cao and Yuan‐Hao Du designed this study. Jiang‐Peng Cao, Xiao‐Xi Liu, Ling‐Fei Wang, Yue‐Xin Lin, and Xu‐Juan Chen performed the study. Jiang‐Peng Cao, Lin Zhang, and Ying Zhou contributed to data analysis. Yuan‐Hao Du supervised the project and acquired funding. Jiang‐Peng Cao, Lin‐Ling Chen, and Xiao‐Xi Liu wrote the manuscript. All authors read and approved the final version of the manuscript.

## Funding

This study was funded by grant 82074543 from the National Natural Science Foundation of China (to Dr. Yuan‐Hao Du), grant JYTC2025022 from the independent research project of First Teaching Hospital of Tianjin University of Traditional Chinese Medicine, and grant tjmzy2401 from the Construction Project of Famous Traditional Chinese Medicine Yuan‐Hao Du Inheritance Studio of China (to Dr. Yuan‐Hao Du).

## Ethics Statement

All patients were informed and signed written informed consent, and the study protocol was reviewed and approved by the Institutional Review Board of the First Teaching Hospital of Tianjin University of Traditional Chinese Medicine (Approved #: TYLL2023[Z]043) and registered at www.chictr.org.cn (ChiCTR2300079204) in December 2023. All procedures using laboratory animals were approved by the Animal Ethics Committee of Tianjin University of Traditional Chinese Medicine (TCM‐LAEC2024020h1555).

## Conflicts of Interest

The authors declare no conflicts of interest.

## Data Availability

The data that support the findings of this study are available on request from the corresponding author.
